# Long-term exposure to elevated carbon dioxide does not alter activity levels of a coral reef fish in response to predator chemical cues

**DOI:** 10.1007/s00265-017-2337-x

**Published:** 2017-07-05

**Authors:** Josefin Sundin, Mirjam Amcoff, Fernando Mateos-González, Graham D. Raby, Fredrik Jutfelt, Timothy D. Clark

**Affiliations:** 1 Department of Neuroscience, Uppsala University, Uppsala, Sweden; 2 Department of Zoology/Functional Zoomorphology, Stockholm University, Stockholm, Sweden; 3 Section of Integrative Biology, University of Texas, Austin, TX USA; 4 Department of Collective Behaviour, Max Planck Institute for Ornithology, University of Konstanz, Konstanz, Germany; 5 Australian Institute of Marine Science, Townsville, Queensland Australia; 6 Great Lakes Institute for Environmental Research, University of Windsor, Windsor, Ontario Canada; 7 Department of Biology, Norwegian University of Science and Technology, Trondheim, Norway; 8 University of Tasmania and CSIRO Agriculture and Food, Hobart, Tasmania Australia

**Keywords:** Climate change, Ocean acidification, Pomacentridae, Olfaction, Alarm cue

## Abstract

**Abstract:**

Levels of dissolved carbon dioxide (CO_2_) projected to occur in the world’s oceans in the near future have been reported to increase swimming activity and impair predator recognition in coral reef fishes. These behavioral alterations would be expected to have dramatic effects on survival and community dynamics in marine ecosystems in the future. To investigate the universality and replicability of these observations, we used juvenile spiny chromis damselfish (*Acanthochromis polyacanthus*) to examine the effects of long-term CO_2_ exposure on routine activity and the behavioral response to the chemical cues of a predator (*Cephalopholis urodeta*). Commencing at ~3–20 days post-hatch, juvenile damselfish were exposed to present-day CO_2_ levels (~420 μatm) or to levels forecasted for the year 2100 (~1000 μatm) for 3 months of their development. Thereafter, we assessed routine activity before and after injections of seawater (sham injection, control) or seawater-containing predator chemical cues. There was no effect of CO_2_ treatment on routine activity levels before or after the injections. All fish decreased their swimming activity following the predator cue injection but not following the sham injection, regardless of CO_2_ treatment. Our results corroborate findings from a growing number of studies reporting limited or no behavioral responses of fishes to elevated CO_2_.

**Significance statement:**

Alarmingly, it has been reported that levels of dissolved carbon dioxide (CO_2_) forecasted for the year 2100 cause coral reef fishes to be attracted to the chemical cues of predators. However, most studies have exposed the fish to CO_2_ for very short periods before behavioral testing. Using long-term acclimation to elevated CO_2_ and automated tracking software, we found that fish exposed to elevated CO_2_ showed the same behavioral patterns as control fish exposed to present-day CO_2_ levels. Specifically, activity levels were the same between groups, and fish acclimated to elevated CO_2_ decreased their swimming activity to the same degree as control fish when presented with cues from a predator. These findings indicate that behavioral impacts of elevated CO_2_ levels are not universal in coral reef fishes.

**Electronic supplementary material:**

The online version of this article (doi:10.1007/s00265-017-2337-x) contains supplementary material, which is available to authorized users.

## Introduction

Ocean acidification, caused by increased levels of dissolved carbon dioxide (CO_2_) (Caldeira and Wickett 2005; Doney et al. 2009), is expected to affect the fitness of marine organisms, especially if CO_2_ levels reach those predicted for the year 2100 in a business-as-usual emission scenario (i.e., ~1000 μatm) (Ishimatsu et al. 2008; Dupont et al. 2010; Heuer and Grosell 2014). The research effort to understand how ocean acidification may affect marine organisms is important and growing rapidly. One increasingly popular topic is the potential for elevated CO_2_ to impact fish behavior. Indeed, the number of papers on ocean acidification and fish behavior almost doubled between 2012 and 2015 (Clements and Hunt 2015; Browman 2016), with the majority of them reporting disturbed behaviors under elevated CO_2_. Given the importance of using appropriate methods to measure seawater pH and the controversy with using pH to estimate CO_2_ (Dickson 1984; Crawford and Harrison 1997; Moran 2014; Bockmon and Dickson 2015), we herein specify the measurement method used for each cited study (where direct measurements of CO_2_ partial pressure (*p*CO_2_) are indicated using μatm, and measurements of pH on the NBS and total scale are indicated using pH_NBS_ and pH_TOTAL_, respectively).

Some studies have suggested that the reported effects of elevated CO_2_ on fish behavior will translate to detrimental effects on fish communities in the wild (reviewed in Heuer and Grosell 2014; Clements and Hunt 2015). For example, studies on adult and larval coral reef fishes report that CO_2_ exposure increases swimming activity in observations performed in situ with the naked eye once fish are placed back into present-day reef conditions (*p*CO_2_ 400–500 μatm) following brief exposure to forecasted CO_2_ conditions (e.g., Ferrari et al. 2011a after 4 days’ exposure to ~850 μatm; Munday et al. 2010 after 4 days’ exposure to 850 μatm; Devine et al. 2012 after 4 days’ exposure to ~960 μatm). In some instances, in which experiments were performed in laboratory settings (manually quantifying activity by counting the number of lines crossed by a fish in an aquarium), CO_2_ exposure has been reported to increase activity levels two-fold (Cripps et al. 2011 4–11 days’ exposure to pH_NBS_ 7.88) or even 90-fold (Munday et al. 2013 4 and 28 days’ exposure to 940 μatm) compared with animals kept at present-day levels of CO_2_. There are a few exceptions, with some studies reporting no effects of elevated CO_2_ on activity (e.g., Ferrari et al. 2012a 4 days’ exposure to pH_NBS_ 7.89; Nowicki et al. 2012 21 days’ exposure to pH_NBS_ 7.89–7.93).

The aspect of fish behavior receiving the most attention with respect to elevated CO_2_ is the response to chemical cues, such as the alarm cues released from conspecifics and/or heterospecifics, or the array of chemical cues emitted from predators (reviewed in Leduc et al. 2013; Clements and Hunt 2015). Perhaps most alarmingly, several studies have reported that prey fish exposed to elevated CO_2_ are attracted to chemical cues from predators rather than avoiding them (Dixson et al. 2010 11 days’ exposure to pH_NBS_ 7.8; Munday et al. 2010 1–10 days’ exposure to 850 μatm; Munday et al. 2012 4 days’ exposure to pH_NBS_ 7.98; Nilsson et al. 2012 4 days’ exposure to pH_NBS_ 7.81; Munday et al. 2013 4 and 28 days’ exposure to 940 μatm). Similarly, several prey species exposed to high CO_2_ have been reported to show a reduced response to chemical alarm cues from the skin of conspecifics (Ferrari et al. 2011a 4 days’ exposure to ~850 μatm; Lönnstedt et al. 2013 4 days’ exposure to pH_NBS_ 7.89; Welch et al. 2014 40–45 days’ exposure to pH_NBS_ 7.85) and an impaired ability to learn to recognize predator chemical cues (Ferrari et al. 2012a 4 days’ exposure to pH_NBS_ 7.89; Chivers et al. 2014 4 days’ exposure to pH_NBS_ 7.85). CO_2_-induced behavioral impairments reportedly also occur in piscivores. For example, brown dottybacks (*Pseudochromis fuscus*) exposed to elevated CO_2_ (pH_NBS_ 7.88) for 4–11 days were reported to be less attracted to prey skin extract compared with control individuals (Cripps et al. 2011), and the elasmobranch smooth dogfish (*Mustelus canis*) was reported to avoid squid chemical cues after a 5-day exposure to pH_NBS_ 7.69 (Dixson et al. 2015).

Given the need to make predictions about the ecological effects of ocean acidification, it is a priority to clarify the behavioral effects in fishes acclimated to elevated CO_2_ (Browman 2016). To help elucidate the effects of long-term exposure to CO_2_ levels predicted for the year 2100 (i.e., ~1000 μatm; Caldeira and Wickett 2005; Doney et al. 2009), we exposed a coral reef damselfish, the spiny chromis (*Acanthochromis polyacanthus*), to elevated CO_2_ for >80 days, before assessing routine activity levels as well as the behavioral response to predator chemical cues. Activity was measured using automated video-tracking software before and after the injection of chemical cues from the flagtail grouper (*Cephalopholis urodeta*), a natural predator of damselfishes on the Great Barrier Reef, Australia (Holbrook and Schmitt 2003). Given the reports of elevated CO_2_ increasing activity in coral reef fishes, with implications for higher predation rates (Munday et al. 2010 1–10 days’ exposure to 850 μatm; Cripps et al. 2011 4–11 days’ exposure to pH_NBS_ 7.88; Devine et al. 2012 4 days’ exposure to ~960 μatm; Munday et al. 2013 4 and 28 days’ exposure to 940 μatm), we predicted that we would similarly detect increased activity in the CO_2_-acclimated fish used in this study. Furthermore, in a previous experiment that investigated transgenerational effects of CO_2_ in *A. polyacanthus*, offspring reared in elevated CO_2_ (at pH_NBS_ 7.85 for 40–45 days) were reported to be strongly attracted to conspecific chemical alarm cues, whereas individuals in control water strongly avoided them (Welch et al. 2014). Based on that study and the current view in the field that the behavioral effects of CO_2_ are strong and highly replicable (reviewed in Clements and Hunt 2015), we predicted that the typical anti-predator behavioral response to chemical cues (a decrease in activity; see Kelley 2008 and references within) would be impaired or reversed. Thus, we predicted that activity levels would be maintained or even increase upon exposure to predator chemical cues in fish acclimated to high CO_2_.

## Methods

### Animals and CO_2_ exposure

Experiments were performed at the Australian Institute of Marine Science (AIMS; Townsville, Australia) between May and July 2015. Juvenile spiny chromis (mean ± SD initial *N* = 753, 0.019 ± 0.015 g initial wet weight, 10.6 ± 2.3 mm initial total length (TL), age ~3–20 days post-hatching) were obtained from the Reef HQ Aquarium in Townsville, Australia. The Reef HQ Aquarium is a large public aquarium, which resembles a natural coral reef environment and requires all small animals to remain vigilant to ensure they are not preyed upon. Clutches of fish were caught across a spatial and temporal scale to ensure they were from at least four breeding pairs. We chose this model species on the basis that it has been reported to suffer dramatic behavioral impairments in response to elevated CO_2_, and it apparently lacks the capacity to reverse these impairments even following transgenerational acclimation (Welch et al. 2014). Notably, previous studies have reported that responses to elevated CO_2_ in captive-bred fishes can be as strong as those of wild-caught fishes (e.g., Munday et al. 2013; Green and Jutfelt 2014; Pimentel et al. 2014; Ou et al. 2015; Tix et al. 2017), including in experiments where the fish had been captive-bred for many generations (Rossi et al. 2015; Pistevos et al. 2017).

Fish were transported in aerated seawater to AIMS where they were placed in twelve 25-L tanks with flow-through seawater (~3.5 L min^−1^) from four independent 200-L sumps (three tanks per sump). After at least 24 h to recover from transport, the partial pressure of CO_2_ (*p*CO_2_) of half of the tanks (*N* = 6) was gradually increased to 1012 ± 137 μatm (mean ± SD) over 24 h using a CO_2_ dosing system (pH stat Computers, Aqua Medic, Bissendorf, Germany) connected to solenoid valves regulating administration of 100% CO_2_ gas into two of the partial-recirculation sump systems. The remaining tanks (*N* = 6) were kept at ambient *p*CO_2_ levels (424 ± 13 μatm). Fresh seawater was flushed through each of the four sumps at ~4–7 L min^−1^. Three air stones in each sump ensured that the water remained well-mixed and maintained dissolved oxygen at >90% air saturation. The *p*CO_2_ levels of the holding tanks were checked every 1–4 days using a LI-820 CO_2_ Gas Analyzer (LI-COR®, Lincoln, Nebraska, USA) (Table 1). Methods for measuring and manipulating *p*CO_2_ thus followed best practice guidelines (Riebesell et al. 2010; Moran 2014; Cornwall and Hurd 2016). Fish were exposed to natural water temperatures for the region (quantified using thermal data-loggers sampling every 30 min; iButton, Maxim Integrated, San Jose, CA, USA). Temperature declined seasonally from 27.0 ± 0.3 °C during the first seven days of holding to 22.1 ± 0.6 °C during the seven days immediately prior to the commencement of the behavioral trials (Table 1). Total alkalinity of the water was measured in duplicate at the mid- and endpoints of the experiment (Table 1). The salinity remained at 35 ± 1 PSU at all times (regulated through the AIMS SeaSim aquarium facility). Water carbonate chemistry was calculated using the constants of Dickson (1990) and Lueker et al. (2000) in CO_2_calc (Hansen, USGS, USA) (Table 1). Fish were fed ad libitum 1–2 times per day using commercial aquaculture pellets crushed to a powder and/or *Artemia* spp. nauplii. Individuals were given 12–18 h without food prior to their use in behavioral experiments to ensure they were in a post-absorptive state (as per Hamilton et al. 2014; Ramasamy et al. 2015). Tanks were cleaned weekly.

**Table 1:** Water chemistry data for the four sump systems (Control 1 and 2; High CO_2_ 1 and 2) which each supplied three holding tanks during the CO_2_ exposure period (May 7–July 27) prior to experiments. *p*CO_2_ was measured every 1–4 days, temperature was logged using iButton data-loggers (one sample per 30 min), and alkalinity was measured on two occasions. pH_TOTAL_ was calculated using CO_2_calc. Data are presented as mean ± SD, and the seasonally dependent range in temperature is given in parentheses

Sump system	*p*CO_2_ (μatm)	Temperature (°C)	Alkalinity (umolkg-1)	pH_tot_ (calc.)
Control 1	418 ± 13	24.5 ± 1.4 (21.4–30.2)	2353 ± 5	8.04 ± 0.00
Control 2	428 ± 13	24.6 ± 1.6 (21.4–30.1)	2363 ± 6	8.03 ± 0.01
*Mean control*	*424 ± 13*	*24.6 ± 1.5*	*2358 ± 8*	*8.03 ± 0.01*
High CO_2_ 1	1028 ± 101	24.4 ± 1.4 (21.3–30.3)	2353 ± 7	7.70 ± 0.03
High CO_2_ 2	1008 ± 144	24.6 ± 1.5 (21.4–29.9)	2356 ± 4	7.71 ± 0.04
*Mean high CO*_*2*_	*1012 ± 137*	*24.5 ± 1.5*	*2355 ± 6*	*7.70 ± 0.04*

### Cue preparation

Water containing predator chemical cues was collected from two tanks (25 L) housing flagtail groupers (*C. urodeta*). Each tank was supplied with water from one of the sump systems (either control or high CO_2_ water) and contained a small, lightly bubbling air stone to ensure that the water remained well-mixed. All effluent water from the predator tanks went straight to the waste drain rather than back to the sump, to ensure that the spiny chromis did not become habituated to predator chemical cues. The tank containing control water housed two groupers with body mass of 50.2 and 61.6 g (4.5 g fish per liter of water) and the tank containing water with elevated *p*CO_2_ housed two groupers with body mass of 33.4 and 78.3 g (4.5 g fish per liter of water). The groupers were exposed to their respective treatment conditions for 11 weeks. The groupers were fed ad libitum 3–4 times per week with whole, freshly sacrificed spiny chromis (sacrificed using cerebral percussion). Each morning of the behavioral experiments (three consecutive days), the flow-through water in the predator tanks was turned off for 3 h (the air stone ensured dissolved oxygen remained >85% air saturation) before 200 mL of predator water was collected from each tank. This protocol was designed to achieve a predator cue concentration comparable to that used in previous studies (Table 2). At the same time, seawater samples without predator cues (sham water) from the control and high CO_2_ treatments were collected from the sumps. All water samples were kept refrigerated throughout the day and used within 8 h. Subsamples of 5 mL, prepared in syringes, were warmed to ambient water temperature over ~90 min prior to use in experiments.

**Table 2:** Summary of methods and details of the preparation of predator chemical cues used in previous studies, focusing on studies of coral reef fishes where the cue was injected directly into the experimental arena. Given are the references (Ref.), predator species (Species), number of individual predators per tank (No. pred.), predator body length (Length), predator biomass (Biomass; see footnote), predator tank water volume (Vol.), calculated predator biomass per liter of water (Rel. biomass), duration that the flow-through was ceased prior to collecting predator water (Time flow off), calculated total cue concentration (Total conc.), method of sample storage (Storage), volume injected in the experimental arena relative to arena volume (Inject: arena), and calculated apparent predator cue concentration (Apparent conc.). Apparent predator cue concentration was calculated using “Rel. biomass,” “Time flow off,” and “Inject: arena” as described in the “Methods” section. Cases where insufficient information was given are denoted NA

Ref.	Species	No. pred.	Length (mm)	Biomass (g)^#^	Vol. (L)	Rel. biomass (g L^−1^)	Time flow off (h)	Total conc. (g L^−1^)	Storage	Inject: arena (mL)	Apparent conc. (g L^−1^)
^‡^	*C. urodeta*	2	143; 152 & 129; 165^T^	111.8 & 111.7	25	4.47 & 4.47	3	13.42 & 13.40	Fridge	5:500	0.13 & 0.13
1	*P. fuscus*	1	57.8 ± 5.6^S^	2.3	6	0.39	96	37.58	Freezer	60:12,000	0.19
2	*P. fuscus*	2	57; 79^S^	2.2; 6.2	10	0.85	6	5.07	Freezer	30:13,000	0.01
	*S. dermatogenys*	2	93; 102^S^	16.0; 21.6	10	3.75	6	22.52	Freezer	30:13,000	0.05
	*C. batuensis*	2	124; 86^S^	42.9; 14.0	10	5.69	6	34.13	Freezer	30:13,000	0.08
3	*C. cyanostigma*	2	270; 250 & 290; 250^T^	488.4 & 554.8	30	16.28 & 18.49	56	911.61 & 1035.57	NA^g^	20:14000^f^	1.44
4	*P. fuscus*	2	65; 71^S^	3.4; 4.5	70	0.11	NA^d^	–	<20 min	20:20,000	–
5	*T. lunare*	1	NA	–	30	–	12	–	NA	NA:15,000	–
	*S. dermatogenys*	1	NA	–	30	–	12	–	NA	NA:15,000	–
6	*C. boenak*	NA	170.2 ± 11.4^S^	134.9	15	–	24	–	<20 min	60:15,000	–
7	*P. fuscus*	≈6–8^a^	NA	–	32	–	NA	–	NA	60:NA	–
8	*P. fuscus*	≈6–8^a^	NA	–	32	–	NA	–	NA	60:NA	–
9	*C. argus*	1	NA	–	70	–	2	–	NA^g^	60:7800	–
10	*P. fuscus*	1	60 & 70^S^	2.6 & 4.3	9	0.29 & 0.47	18:00—O/N	3.52 & 5.68^e^	NA^g^	20:9000	0.01 & 0.01
11	*T. lunare*	≥2^b^	NA	–	Adj.	1.1^c^	O/N	13.20^e^	NA	30:13,000	0.03
	*T. hardwicke*	≥2^b^	NA	–	Adj.	1.1^c^	O/N	13.20^e^	NA	30:13,000	0.03
	*C. batuensis*	≥2^b^	NA	–	Adj.	1.1^c^	O/N	13.20^e^	NA	30:13,000	0.03
	*P. fuscus*	≥2^b^	NA	–	Adj.	1.1^c^	O/N	13.20^e^	NA	30:13,000	0.03
12	*T. lunare*	6	NA	–	20	–	2	–	NA	30:20,000	–
	*S. dermatogenys*	4	NA	–	20	–	2	–	NA	30:20,000	–
	*P. fuscus*	4	NA	–	20	–	2	–	NA	30:20,000	–
13	*P. fuscus*	1	100^T^	6.5	10	0.65	12	7.74	<1 day	2:10	1.55
14	*T. lunare*	≥2^b^	NA	–	Adj.	1.1^c^	O/N	13.20^e^	NA^g^	30:13,000	0.03
	*T. hardwicke*	≥2^b^	NA	–	Adj.	1.1^c^	O/N	13.20^e^	NA^g^	30:13,000	0.03
	*C. batuensis*	≥2^b^	NA	–	Adj.	1.1^c^	O/N	13.20^e^	NA^g^	30:13,000	0.03
	*P. fuscus*	≥2^b^	NA	–	Adj.	1.1^c^	O/N	13.20^e^	NA^g^	30:13,000	0.03
15	*P. fuscus*	NA	74.2 ± 0.9^S^	5.1	32	–	16	–	NA	30:32,000	–

Genus and species names *Cephalopholis urodeta*, *Pseudochromis fuscus*, *Synodus dermatogenys*, *Coris batuensis*, *Cephalopholis cyanostigma*, *Thalassoma lunare*, *Cephalopholis boenak*, *Cephalopholis argus*, *Talassoma hardwicke*

*Superscripted S* standard length, *T* total length

*Adj.* adjusted by authors to reach targeted “concentration” of 1.1 g L^−1^ water

*O/N* stated by authors as “overnight” or as “18:00 and overnight”

*1* Holmes and McCormick (2010); *2 *Mitchell et al. (2011); *3* Bosiger et al. (2012); *4* Ferrari et al. (2012a); *5* Lönnstedt et al. (2012); *6* Manassa and McCormick (2012); *7* Manassa and McCormick (2013); *8* Manassa et al. (2013a); *9* Manassa et al. (2013b); *10* Mitchell and McCormick (2013); *11* Mitchell et al. (2013); *12* Chivers et al. (2014); *13* Atherton and McCormick (2015); *14* Mitchell et al. (2015); *15* Ramasamy et al. (2015)

^‡^Present study

^#^Measured in the present study but calculated for all other studies based on length-mass relationships in Froese et al. (2014) and adjusting standard length to total length, where necessary, using a factor of 1.25

^a^Stated in the papers as “approximately 6–8”

^b^Stated in the papers as “at least two”

^c^Predator mass per liter specified in the paper

^d^Authors stated 60% of the water volume was changed per day

^e^Assuming that the author statements “18:00 and overnight” and “overnight” are equivalent to 12 h

^f^Stated that 10 mL from each of two predator tanks was injected into a 14-L experimental arena, which gives one apparent predator cue concentration

^g^Details not provided in original reference but stated as “consistently available,” “collected just prior to experiment,” “collected prior to start,” or “used the following morning”

### Experimental design

Following 80–82 days of exposure to present-day or elevated CO_2_ (on July 25–27, 2015), we commenced experiments to investigate whether spiny chromis exposed to high CO_2_ differed from control fish in their routine activity levels as well as in their behavioral response to predator chemical cues. We did this by measuring routine activity levels of individual fish before and after the injection of sham/predator water. Nine white opaque plastic arenas (diameter 11 cm; water volume 500 mL) containing water (23.3 ± 0.27 °C, >98% air saturation) of the appropriate exposure treatment were placed in a 3 × 3 arrangement (Electronic Supplementary Material, Fig. S1), in a shallow reservoir aquarium receiving flow-through water to maintain temperature. The location of high CO_2_ and control fish within the 3 × 3 arena grid was randomized for each trial. Each arena was fitted with an ~80 cm length of thin silicone tubing, one end of which was glued vertically to the inside of the arena such that it extended 1 cm below the surface of the water (Electronic Supplementary Material, Fig. S1). The other end of the tube was anchored ~20 cm outside the reservoir aquarium to enable the injection of cue water (seawater with or without predator cues). A curtain surrounding the reservoir aquarium minimized visual disturbances and prevented the fish from seeing the experimenter, including during the injection of sham/predator water.

The spiny chromis (TL mean ± SD CO_2_ 42.7 ± 4.7 mm, control 41.6 ± 4.0 mm; two-sample *t* test *t*_71_ = 1.07; *P* = 0.288) were placed individually into the experimental arenas and left undisturbed in moderate ambient light. After 90 min, 5 mL of sham water or 5 mL of predator cue water (Table 2) was slowly injected into each arena using 50-mL syringes connected to the end of the tubes extending outside the reservoir aquarium. All arenas were injected with cue within a 2-min period, and the fish were then left undisturbed for an additional 30 min. The fish in all nine arenas were monitored simultaneously throughout the experimental period using a FireWire camera (Dragonfly 2, Point Gray, Richmond, BC, Canada) mounted approximately 1 m above the arenas. To minimize observer bias, blinded methods were used when extracting and analyzing all behavioral data from the footage. We used tracking software (ViewPoint Zebralab; ViewPoint, Lyon, France) to automatically quantify swimming distance and duration (per minute) of each individual fish in real-time (as per Sundin and Jutfelt 2016) for 30 min prior to cue injection and 30 min after cue injection. Based on pilot runs, we iteratively set the swimming threshold to all movements exceeding 0.5 body lengths per second (BL s^−1^). The remaining time reflects time spent inactive (i.e., “swimming duration” is the inverse of “inactivity duration”). The use of tracking software requires high contrast between the fish and the background, as documented in several previous studies that have used similar approaches to quantify behavioral responses, including in the context of elevated CO_2_ (Bignami et al. 2013, 2014; Maneja et al. 2013; Hamilton et al. 2014; Maneja et al. 2015; Ou et al. 2015; Duteil et al. 2016; Sundin and Jutfelt 2016). The protocol was replicated eight times (trials), always using new fish, and randomizing exposure treatments and sham/predator water injections. The following sample sizes were obtained: *N* CO_2_-sham water injection = 16; CO_2_-predator cue injection = 21; Control-sham water injection = 16; Control-predator cue injection = 19. After each trial, the fish were measured for TL and returned to labeled holding tanks. Dissolved oxygen of the water in the arenas declined to a minimum of 76.7 ± 4.4% of air saturation by the end of each trial.

### Statistical analysis

We tested whether there was an effect of CO_2_ treatment on routine activity (i.e., activity levels before cue injection) using analysis of variance (ANOVA). Mean swimming duration and distance (i.e., all movements exceeding 0.5 BL s^−1^) for the 30-min period following acclimation were used as the response variables (i.e., duration and distance, respectively), while treatment (CO_2_ or control) was used as a fixed effect. Fish length was initially included as a covariate, but it was found to have no effect on swimming activity (Length_[duration]_ *F*_1, 64_ = 2.59, *P* = 0.113; Length_[distance]_: *F*_1, 64_ = 0.29, *P* = 0.595) and was therefore removed from the models and is not discussed further.

The predator cue concentration injected into the experimental arenas was estimated for the present and previous studies (where sufficient information was provided, and/or by inferring data) in order to enable comparisons across studies (Table 2). Calculations were conducted as follows (with values from the present study given in parentheses as an example). Predator biomass in the predator holding tanks was established (112 g/25 L = 4.5 g L^−1^) and multiplied by the duration for which the flow-through water was ceased prior to obtaining a water sample (4.5 g L^−1^ × 3 h = 13.4 g L^−1^). This total concentration was multiplied by the amount of cue injected into the experimental arena (13.4 g L^−1^ × 0.005 L = 0.067 g) and then divided by the volume of the experimental arena (0.067 g/0.5 L = 0.13 g L^−1^) to provide an estimate that is herein referred to as the “apparent predator cue concentration.” In instances where only predator length was provided in previous studies, we estimated mass based on length-mass relationships in Froese et al. (2014), after adjusting standard length to total length using a factor of 1.25 where necessary. While these estimates contain several assumptions (e.g., that predator cue increases linearly over time upon cessation of flow-through water) and do not represent true concentrations, they provide useful information for comparing across studies and highlighting some of the critical details that we urge authors to include in future papers in this field (Table 2).

To investigate the response to sham/predator cues, we calculated the difference in mean swimming duration and distance between the pre- and post-injection periods (i.e., mean duration and distance during the 30-min pre-injection period subtracted from mean duration and distance during the 30-min post-injection period; 2 min was excluded between these periods to allow for cue injections). This analytical approach accommodates for inter-individual variation in fish activity levels and allows for easier comparisons with previous studies that have used a similar approach (e.g., Ferrari et al. 2011a, b, 2012a, b; Lönnstedt et al. 2013; Chivers et al. 2014). Differences in mean swimming duration and distance were the response variables in ANOVAs, whereas treatment (CO_2_ or control), cue type (sham water or predator water), and their interaction were included as fixed effects. Since the predator cue was prepared in the morning of each day and refrigerated for use throughout the day, we further included time of day as a factor, and the interaction between time of day and cue type, to investigate possible degradation of the predator cue. Fish length was again included as a covariate and had no effect on swimming activity (Length_[duration]_ *F*_1, 57_ = 0.80, *P* = 0.374; Length_[distance]_ *F*_1, 57_ = 1.45, *P* = 0.234) and was therefore removed from the models. For the minimal-adequate model, non-significant interactions (*α* level 0.10) were dropped using stepwise backward exclusion. Dropped interactions were again included in the final model, one at a time, to verify that they did not have a significant effect. Since none of the interactions were found to be significant (*P* > 0.30), the final model contained cue, treatment, and time of day (final model ANOVA, duration *F*_6, 56_ = 2.81, *P* = 0.019, adjusted *R* ^2^ = 0.15, ΔAIC_c_ = 8 compared with full model; distance *F*_6, 56_ = 3.17, *P* = 0.010, adjusted *R* ^2^ = 0.17, ΔAIC_c_ = 7 compared with full model). In all cases, the data met the assumptions of residual normal distribution and homogeneity of variance (Shapiro-Wilk *W* > 0.9, *P* > 0.50, Levene *F*_1_ > 0.2, *P* > 0.20, respectively). JMP 11 (SAS Institute Inc., Cary, NC, USA) was used for the statistical analyses.

## Results

**Fig. 1:**
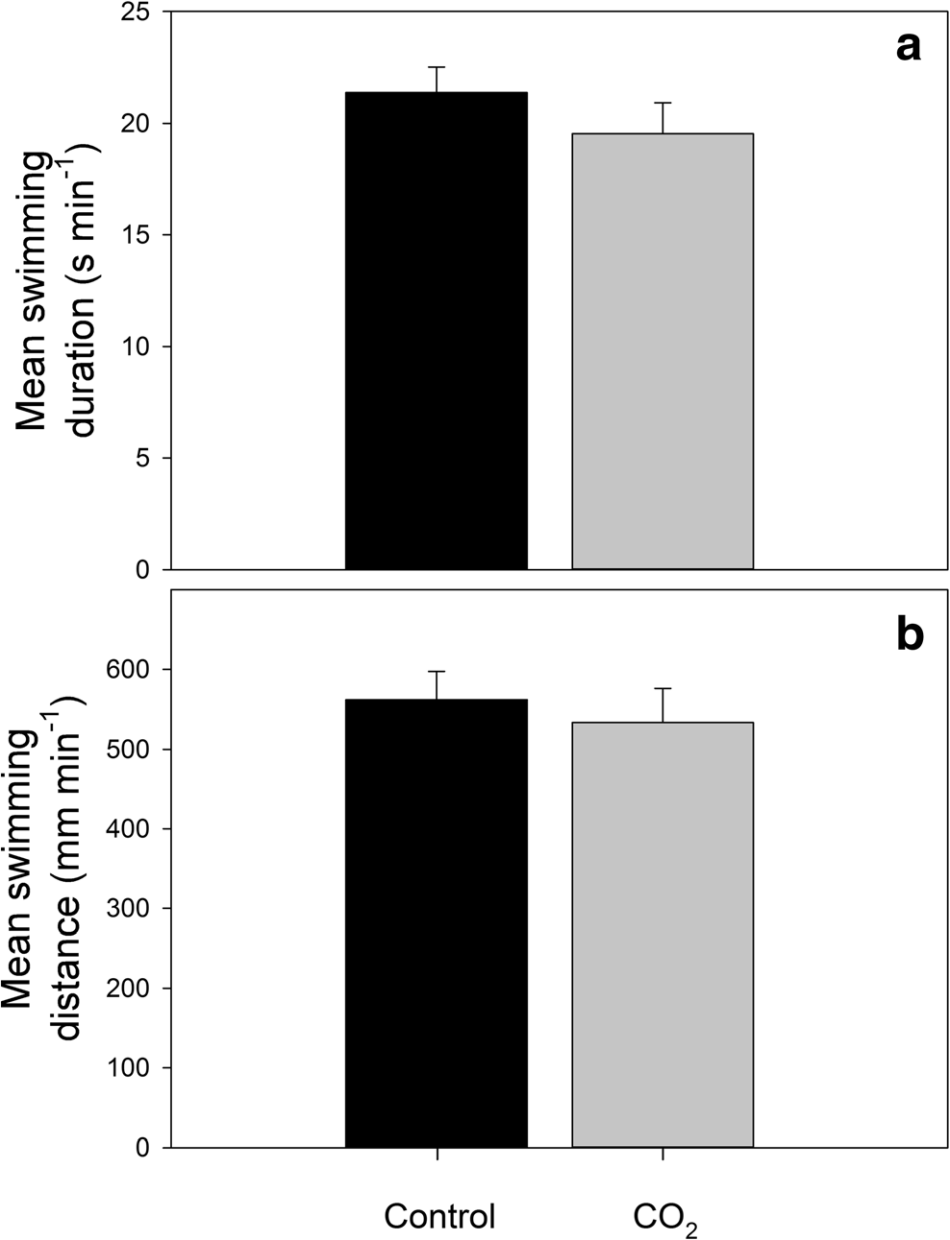
Mean ± SE routine swimming duration (**a**) and swimming distance (**b**) of juvenile spiny chromis (*Acanthochromis polyacanthus*) after being reared for 3 months in control (~420 μatm, *black bars*) or high CO_2_ (~1000 μatm, *gray bars*) treatments at 23.3 ± 0.27 °C. Values were calculated during the 30-min period prior to cue injection

In contrast with our prediction, there were no differences in routine activity levels between CO_2_ treatment groups during the 30-min period prior to injection of the cue (Treatment_[duration]_ *F*_1, 65_ = 1.27, *P* = 0.264; Treatment_[distance]_ *F*_1, 65_ = 0.30, *P* = 0.588; Fig. 1). Combining both CO_2_ treatment groups and both cue type groups (sham and predator cue), fish spent 20.4 ± 0.9 s min^−1^ swimming at >0.5 BL s^−1^ (mean ± S.E.), and they swam a distance of 547 ± 28 mm min^−1^ during the 30 min prior to cue injection (Fig. 1).

**Fig. 2:**
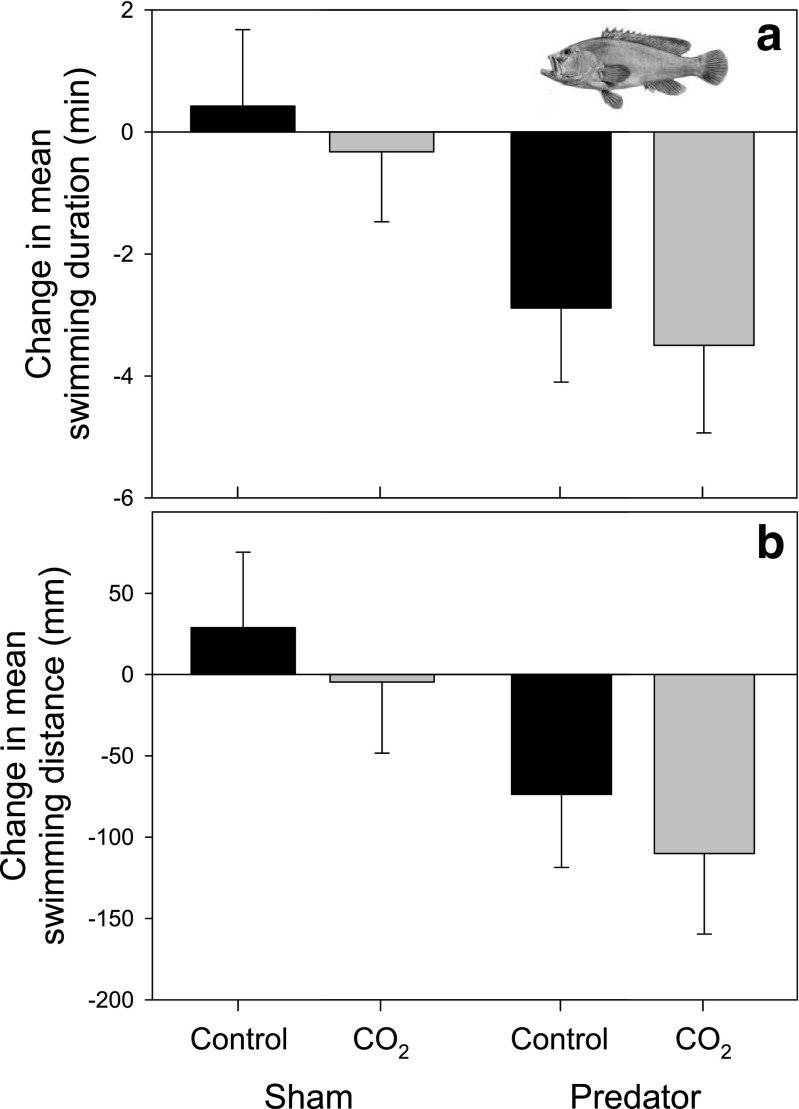
Mean ± SE change in swimming duration (**a**) and swimming distance (**b**) of juvenile spiny chromis (*Acanthochromis polyacanthus*) between the 30-min pre- and post-injection periods, where injections were sham (non-predator) water, or water-containing predator cues. Fish had been reared for 3 months in control (~420 μatm, *black bars*) or high CO_2_ (~1000 μatm, *gray bars*) treatments at 23.3 ± 0.27 °C (inserted illustration: flagtail grouper, *Cephalopholis urodeta*; George Henry Ford, https://commons.wikimedia.org/wiki/File%3ACephalopholis_urodeta.jpg)

While comparisons of predator cue “concentrations” across studies are difficult due to a general lack of details (Table 2), through our calculations of apparent predator cue concentrations, we established that the levels we used were at least as high as those estimated for the majority of previous studies (0.13 g L^−1^; Table 2). As predicted, cue type had an effect on the swimming duration and distance of spiny chromis, whereby activity decreased in fish receiving the predator cue injection but did not differ from pre-injection levels in fish receiving the sham water injection (Cue type_[duration]_ *F*_1, 64_ = 7.88, *P* = 0.007; Cue type_[distance]_ *F*_1, 64_ = 6.42, *P* = 0.014; Fig. 1). This response was not dependent on CO_2_ treatment, since the cue-induced changes in swimming duration and distance were the same in the control and high CO_2_ groups (Treatment_[duration]_ *F*_1, 64_ = 0.14, *P* = 0.709; Treatment_[distance]_ *F*_1, 64_ = 0.48, *P* = 0.491; Fig. 2). Pooling data from both CO_2_ treatment groups, mean swimming duration after the predator cue injection was 85% of the duration before the injection (mean ± SE duration before 21.0 ± 1.2 s min^−1^, duration after 17.8 ± 1.1 s min^−1^) and swimming distance after predator cue injection was 83% of the distance before the injection (mean ± SE distance before 553 ± 39 mm min^−1^, distance after 460.4 ± 32.0 mm min^−1^).

Time of day had a significant effect on the mean difference in activity pre- vs. post-injection (Time of day_[duration]_ *F*_5, 64_ = 4.57, *P* = 0.001; Time of day_[distance]_ *F*_5, 64_ = 5.11, *P* = 0.001). However, the lack of an interaction between cue type and time of day (Time of day ∗ Cue type_[duration]_ *F*_5, 59_ = 0.71, *P* = 0.621; Time of day ∗ Cue type_[distance]_ *F*_5, 59_ = 1.28, *P* = 0.283) suggests that the time of day effect was unrelated to sequential changes in the predator chemical cue during refrigeration and re-warming. This lack of interaction also suggests that fish were not responding differently as the day progressed (e.g., due to potentially increased hunger levels). Indeed, Tukey’s HSD post-hoc tests revealed that one specific trial (trial 7 on day 2 (July 26)), rather than a specific time of day, elicited the significant effect of time of day. Excluding that trial confirmed our initial results of an effect of cue type on the difference between pre- vs. post-injection activity levels (Cue type_[duration]_ *F*_1, 60_ = 8.67, *P* = 0.005; Cue type_[distance]_ *F*_1, 60_ = 8.47, *P* = 0.005). All other factors, including time of day and interactions, remained non-significant. Time of day had a similar effect on routine activity levels (pre-stimulus activity) (Time of day_[duration]_ *F*_5, 65_ = 2.41, *P* = 0.046; Time of day_[distance]_ *F*_5, 65_ = 2.86, *P* = 0.021), but again this was caused by lower activity in the same trial as indicated above. Correspondingly, CO_2_ treatment remained non-significant in the analysis of pre-stimulus routine activity levels when excluding the trial with anomalously low activity (Treatment_[duration]_ *F*_1, 61_ = 2.15, *P* = 0.148; Treatment_[distance]_ *F*_1, 61_ = 0.70, *P* = 0.405).

## Discussion

Several aspects of predator avoidance behavior have been reported to be negatively affected by increased levels of CO_2_. These include loss of behaviors typically triggered by visual and auditory predator stimuli (Simpson et al. 2011; Ferrari et al. 2012b; Lönnstedt et al. 2013), and attraction to, rather than avoidance of, predator chemical cues (Dixson et al. 2010; Munday et al. 2010, 2012, 2013; Nilsson et al. 2012). Impaired predator avoidance should result in reduced survival in fishes exposed to high CO_2_, which has indeed been reported in experiments in which coral reef fish were exposed to elevated CO_2_ for 4–12 days and then tested in control water (Munday et al. 2010; Ferrari et al. 2011a, b; Munday et al. 2012; Chivers et al. 2014). In addition, increased levels of CO_2_ have been reported to increase activity in several species of coral reef fishes (Munday et al. 2010, 2013; Cripps et al. 2011; Devine et al. 2012) with some exceptions (Ferrari et al. 2012a; Nowicki et al. 2012). Non-coral reef fishes appear to be more robust, as several papers have reported no effect of elevated CO_2_ on activity in a range of species (e.g., Bignami et al. 2013, 2014; Maneja et al. 2013, 2015; Hamilton et al. 2014; Sundin and Jutfelt 2016).

The present study contrasts with the majority of reports on coral reef fishes by showing no effect of elevated CO_2_ on routine activity levels or on the behavioral response to predator cues. Specifically, fish exposed to high CO_2_ for 80–82 days had a similar routine activity level as control fish reared at present-day CO_2_ levels, and they decreased their activity level to the same extent as the control fish when exposed to predator chemical cues. The decreased duration spent swimming after the predator cue injection indicates that the fish spent more time moving slowly (<0.5 BL s^−1^) and/or being immobile, which is a typical response to predator cues (Kelley 2008 and references therein). While a 15% reduction in swimming duration and 17% reduction in swimming distance may seem relatively small, it has been reported that damselfish larvae, *Pomacentrus wardi*, exposed to 850 μatm for 4 days and subsequently transplanted to patch reefs in situ suffer nine-fold higher predation mortality as a consequence of having 24% higher activity levels (~0.6 cm min^−1^ higher) and moving an average of 0.8 cm further from shelter than control larvae (Munday et al. 2010).

The discrepancy between our findings and the majority of previous reports on tropical damselfishes, including *A. polyacanthus*, could be due to several factors, including differences in experimental design, exposure times, the high precision and low vulnerability to biases in the present study due to the use of video-tracking software (Egan et al. 2009; Holman et al. 2015), differences in behavioral responses between and within species (Clements and Hunt 2015), and/or publication and reporting biases (i.e., the observation that studies reporting negative results are typically more difficult to publish) (Browman 1999; Dwan et al. 2013). We expand on some of these points below.

To the best of our knowledge, the present study is the first to use automated tracking software to quantify the activity responses of a coral reef fish to predator chemical cues in the context of ocean acidification (although several studies have examined different contexts and species; e.g., Bignami et al. 2013, 2014; Hamilton et al. 2014; Sundin and Jutfelt 2016). Previous studies on coral reef fishes have typically assessed activity by manually scoring, in real-time with the naked eye, the number of lines crossed by a fish in an aquarium (Ferrari et al. 2011a, 2012a; Nowicki et al. 2012; Lönnstedt et al. 2013; Munday et al. 2014). While not diminishing the value of those studies, video tracking provides more precise data of higher validity and objectivity (Egan et al. 2009). The use of automated video tracking ensures that the scoring of activity is not subject to observer biases (e.g., confirmation bias, which is the unintentional preference to detect and focus on outcomes that confirm prior beliefs; Nickerson 1998; Holman et al. 2015), and it provides a database of visual evidence.

There are additional methodological differences between our study and previous studies examining the effect of CO_2_ on fish behavior. For example, several previous studies have investigated activity levels and responses to chemical cues in combination with a feeding event (Ferrari et al. 2011a, 2012b; Chivers et al. 2014). Although fish were not fed during the behavioral observations in our experiment, an effect of the predator cue was detected, so we do not view this as a confounding factor. Indeed, CO_2_ treatment effects on swimming activity have been reported in previous studies in which food was withheld during behavioral observations (e.g., Munday et al. 2013, 2014; Pimentel et al. 2014; Rossi et al. 2015). We also used a relatively small behavioral arena in relation to fish size compared with some previous studies (e.g., Ferrari et al. 2011a, b), which may have restricted the magnitude of the change in activity of the fish in response to the predator cue. Whether a larger arena would have allowed for a greater reduction in movement following cue injection is unclear. Nevertheless, despite the smaller arenas, the reaction by the fish in our experiment was the same regardless of CO_2_ acclimation treatment. Furthermore, in this study, we used captive-bred fish (from a public aquarium), similar to some previous studies (e.g., Rossi et al. 2015; Pistevos et al. 2017) but in contrast with other studies that have used wild fish or fish 1–2 generations removed from the wild (e.g., Munday et al. 2010). Based on existing literature, the reported effects of elevated CO_2_ do not seem to differ between wild and captive-bred fish, yet clarity on this point must await further research.

It could be argued that the absence of a CO_2_ treatment effect in the present study is due to *A. polyacanthus* being more resilient to high CO_2_ compared with other coral reef damselfishes for which dramatic behavioral impairments have been reported (reviewed in Clements and Hunt 2015). Indeed, Munday et al. (2011) reported no effects of CO_2_ on juvenile growth, survival, skeletal development, or otolith morphometrics after rearing newly hatched *A. polyacanthus* for 21 days in pH_NBS_ 7.83. There also appears to be no detrimental effect on the oxygen uptake rates of this species following 17 days in pH_NBS_ 7.87 (Rummer et al. 2013). However, evidence against the idea of *A. polyacanthus* being resilient to high CO_2_ stems from other studies of this species that have reported profound effects. For example, Chung et al. (2014) reported an altered retinal response in this species after 6–7 days of exposure to pH_NBS_ 7.87. Furthermore, Welch et al. (2014) reported that 40–45 days of exposure to pH_NBS_ 7.85 resulted in reduced behavioral lateralization and attraction to (rather than avoidance of) chemical alarm cues extracted from the skin of conspecifics. Disparate findings within a species have also been reported for the tropical damselfish *P. wardi*. For example, Bender et al. (2015) reported that fish did not show any visible changes in behavior and did not change their food selectivity when exposed to pH_TOTAL_ 7.85 in combination with warming (+4.1 °C) for 4 weeks. Other studies on the same species following 4–7 days of exposure to similar CO_2_ levels reported that CO_2_ exposure induces riskier behavior, causes attraction to predator cues (93% of time spent in predator cues), increases activity, and shifts behavioral lateralization from a right to a left bias (Munday et al. 2010 *p*CO_2_ 850 μatm; Munday et al. 2012 pH_NBS_ 7.98; and Domenici et al. 2014 pH_NBS_ 7.87). Hence, it seems that elevated CO_2_ may or may not cause impairments within a species depending on which behavioral and physiological traits are investigated, but this is a difficult conclusion to make in the absence of standardized replication studies.

In sum, we found that long-term exposure to elevated CO_2_ did not alter routine activity levels or the behavioral response to predator chemical cues in *A. polyacanthus*. This study thus adds to the growing literature reporting no behavioral effects in fishes following acclimation to elevated CO_2_ (Browman 2016). Contrasting findings among studies using the same species demonstrate the importance of replication as a prerequisite to establishing a consensus on how fish behavior may be affected by ocean acidification. Achieving such replication will rely on transparency and the use of robust and standardized methods.

### Data availability

The dataset supporting this manuscript is available as electronic supplementary material and publicly archived in the repository figshare, following best practices (Roche et al. 2015). https://figshare.com/s/64d7082241deda6d4bd4

## Electronic supplementary material

ESM 1 (PDF 1781 kb)

ESM 2 (XLS 4492 kb)
