# Supplementary material for: Long-term exposure to elevated carbon dioxide does not alter activity levels of a coral reef fish in response to predator chemical cues
Source: Behav Ecol Sociobiol. 2017 Jul 5;71(8):108. doi: 10.1007/s00265-017-2337-x (PMC5498585; doi:10.1007/s00265-017-2337-x)
Supplement: Supplementary file 1 — (PDF 1781 kb) [file 265_2017_2337_MOESM1_ESM.pdf]

Electronic Supplementary Material

Long-term exposure to elevated carbon dioxide does not alter activity levels of a coral reef fish in response to predator chemical cues

Behavioral Ecology and Sociobiology

Josefin Sundin*, Mirjam Amcoff, Fernando Mateos-González, Graham D Raby, Fredrik Jutfelt, Timothy D Clark

*Corresponding author:
josefin.sundin@neuro.uu.se; josefin@teamsundin.se
Department of Neuroscience, Uppsala University, Uppsala, Sweden

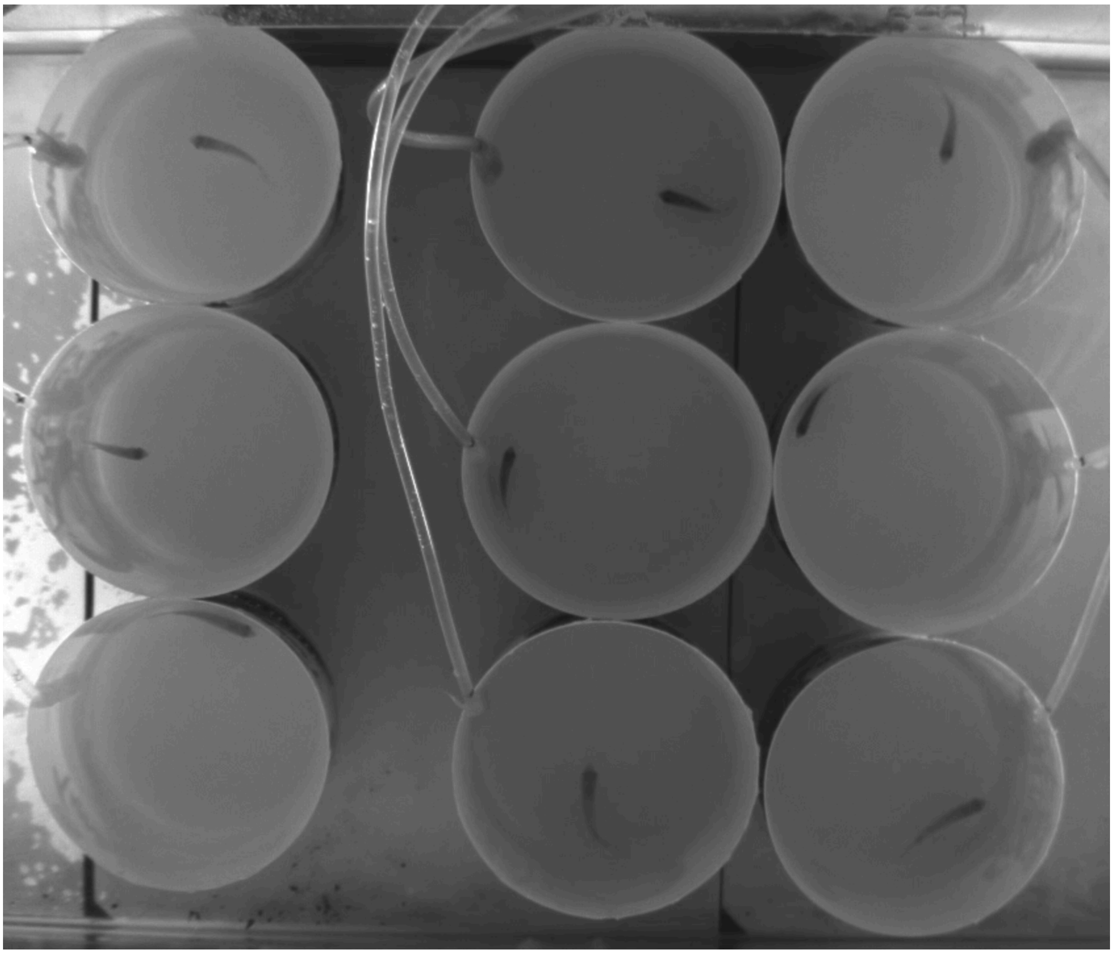

Fig. S1 Overhead image of fish in the nine activity arenas (diameter: 11 cm, water volume: 500 mL) as seen through the camera used to record the behavior of the animals

The electronic supplementary material contains an Excel file with raw data.
